# Postnatal meningeal CSF transport is primarily mediated by the arachnoid and pia maters and is not altered after intraventricular hemorrhage-posthemorrhagic hydrocephalus

**DOI:** 10.1186/s12987-023-00503-7

**Published:** 2024-01-08

**Authors:** Shelei Pan, Joshua P. Koleske, Gretchen M. Koller, Grace L. Halupnik, Abdul-Haq O. Alli, Shriya Koneru, Dakota DeFreitas, Sruthi Ramagiri, Jennifer M. Strahle

**Affiliations:** Department of Neurosurgery, Washington University School of Medicine, Washington University in St. Louis, St. Louis, MO 63110 USA

## Abstract

**Background:**

CSF has long been accepted to circulate throughout the subarachnoid space, which lies between the arachnoid and pia maters of the meninges. How the CSF interacts with the cellular components of the developing postnatal meninges including the dura, arachnoid, and pia of both the meninges at the surface of the brain and the intracranial meninges, prior to its eventual efflux from the cranium and spine, is less understood. Here, we characterize small and large CSF solute distribution patterns along the intracranial and surface meninges in neonatal rodents and compare our findings to meningeal CSF solute distribution in a rodent model of intraventricular hemorrhage-posthemorrhagic hydrocephalus. We also examine CSF solute interactions with the tela choroidea and its pial invaginations into the choroid plexuses of the lateral, third, and fourth ventricles.

**Methods:**

1.9-nm gold nanoparticles, 15-nm gold nanoparticles, or 3 kDa Red Dextran Tetramethylrhodamine constituted in aCSF were infused into the right lateral ventricle of P7 rats to track CSF circulation. 10 min post-1.9-nm gold nanoparticle and Red Dextran Tetramethylrhodamine injection and 4 h post-15-nm gold nanoparticle injection, animals were sacrificed and brains harvested for histologic analysis to identify CSF tracer localization in the cranial and spine meninges and choroid plexus. Spinal dura and leptomeninges (arachnoid and pia) wholemounts were also evaluated.

**Results:**

There was significantly less CSF tracer distribution in the dura compared to the arachnoid and pia maters in neonatal rodents. Both small and large CSF tracers were transported intracranially to the arachnoid and pia mater of the perimesencephalic cisterns and tela choroidea, but not the falx cerebri. CSF tracers followed a similar distribution pattern in the spinal meninges. In the choroid plexus, there was large CSF tracer distribution in the apical surface of epithelial cells, and small CSF tracer along the basolateral surface. There were no significant differences in tracer intensity in the intracranial meninges of control vs. intraventricular hemorrhage-posthemorrhagic hydrocephalus (PHH) rodents, indicating preserved meningeal transport in the setting of PHH.

**Conclusions:**

Differential CSF tracer handling by the meninges suggests that there are distinct roles for CSF handling between the arachnoid-pia and dura maters in the developing brain. Similarly, differences in apical vs. luminal choroid plexus CSF handling may provide insight into particle-size dependent CSF transport at the CSF-choroid plexus border.

**Supplementary Information:**

The online version contains supplementary material available at 10.1186/s12987-023-00503-7.

## Introduction

The role of the meninges in CSF circulation has historically been a subject of longstanding speculation. Nineteenth century French physiologist Francois Magendie, who discovered the eponymous foramen of Magendie, postulated that the leptomeninges (pia and arachnoid mater) produce CSF [1, 2]. Even though this theory was later disregarded due to histologic, pharmacologic, and physiologic studies supporting the choroid plexus (ChP) as the cite of CSF production [3–8], his discoveries highlighted that the meninges are more than a protective barrier. Our understanding of the structural and functional complexities of the meninges has expanded with more recent investigations elucidating specific roles the meninges actively take in the development, maintenance, and function of the CNS. In fetal development, the meninges play an important role in releasing diffusible factors which influence the proliferation and migration of brain parenchyma and epithelial cells [9]. In the adult rodent CNS, the meningeal lymphatics within the dorsal convexities and at the base of the skull play a role in CNS immune surveillance and CSF solute handling [10, 11] alongside  other routes of CSF drainage including CSF efflux along the perivascular and perineural subarachnoid spaces (SAS) into the extracranial lymphatics of the skull base [12]. The leptomeninges and dura mater have different transcriptional signatures and play distinct roles within the CNS [13]. Beyond their spatial heterogeneity, meningeal fibroblasts also have distinct transcriptional signatures across development [13], however it is not clear if meningeal CSF solute handling during development differs from what is observed in adult and aged animals. Additionally, it is not clear if there are differences in CSF handling within the layers of the neonatal meninges including the arachnoid and pia (vs. dura). As there is a complex relationship between the meninges and CSF, it is important to understand how and where in the postnatal meninges CSF is handled.

Particularly relevant to the neonatal and postnatal time period, understanding how the developing meninges handle CSF may also have implications for neurological sequelae after devastating pathologies like neonatal intraventricular hemorrhage-posthemorrhagic hydrocephalus (IVH-PHH). IVH in neonates most commonly occurs as a result of bleeding from the germinal matrix and can cause brain injury and PHH [14]. While PHH has previously been hypothesized to alter intraventricular CSF dynamics, it is unknown how IVH-PHH affects meningeal CSF handing. As many intracranial cisterns are lined by the leptomeninges and adjacent neurodevelopmentally vital structures like the hippocampus and brainstem, understanding postnatal meningeal CSF handling may help provide insight into how neurotoxic blood breakdown products released into the CSF spaces after IVH-PHH may be transported beyond the ventricular system to the developing brain.

In the present study, we present a CNS-wide characterization of CSF solute movement within the meninges and its related structures in neonatal rodents, with special emphasis on the intracranial meninges, spinal meninges, and ChP. Using intraventricular injections of large (15-nm) and small (1.9-nm and 3 kDa) CSF tracers, we show that both large and small CSF tracers distribute primarily within the cranial and spine leptomeninges with more limited distribution within the dura. Both large and small CSF tracers circulate through the leptomeninges of the perimesencephalic cisterns and are also present in the third ventricular tela choroidea and velum interpositum, a distribution pattern that is not affected by IVH-PHH. In the ChP, small CSF tracers are found in the basolateral surface of the ChP epithelial cells, while large CSF tracers preferentially accumulate on the apical surface. Finally, meningeal transport is preserved early after PHH and may act as a route to transmit both physiologic CSF solutes and blood breakdown products after IVH.

## Methods

### Animals (Rodents)

All experiments were approved by the Institutional Animal Care and Use Committee of Washington University (protocol #22-0614). Sprague Dawley Rats (crl:SD400, Charles River Laboratories, Wilmington, MA) were used in all experiments. Female and male post-natal day 4–7 Sprague Dawley Rats were housed with their dams in a 12-h light–dark cycle in a temperature and humidity-controlled room. Water and food were provided ad libitum for the dam.

### Intraventricular hemorrhage-posthemorrhagic hydrocephalus induction

P4 rodents were anesthetized (isoflurane 2–3% induction and 1.5% maintenance) and fixed in a stereotaxic frame. A 2.5 mm midline incision was made and a 0.3 cc syringe with a 30-gauge needle was inserted into the right lateral ventricle (LV) at the following coordinates from bregma: 1.4 mm lateral, 0.5 mm anterior, and 2.0 mm deep. A small volume (20 μL) of artificial CSF (aCSF) (Tocris Bioscience, Bristol, UK) or hemoglobin constituted in aCSF at a 150 mg/mL concentration was injected at a rate of 8000 nL/min using a micro-infusion pump (World Precision Instruments, Sarasota, FL) to create the aCSF control and IVH-PHH conditions respectively. The needle was left in for 5 min post injection to prevent backflow. The incision was closed with 6–0 Ethilon suture (Ethicon Inc, Raritan, NJ). Rodents recovered from anesthesia and were returned to their cage with the dam for 72 h before CSF tracer injection at P7. Rats injected with hemoglobin developed ventriculomegaly by the time of tracer injection at P7 [15–17].

### CSF tracer injections

**Fig. 1:**
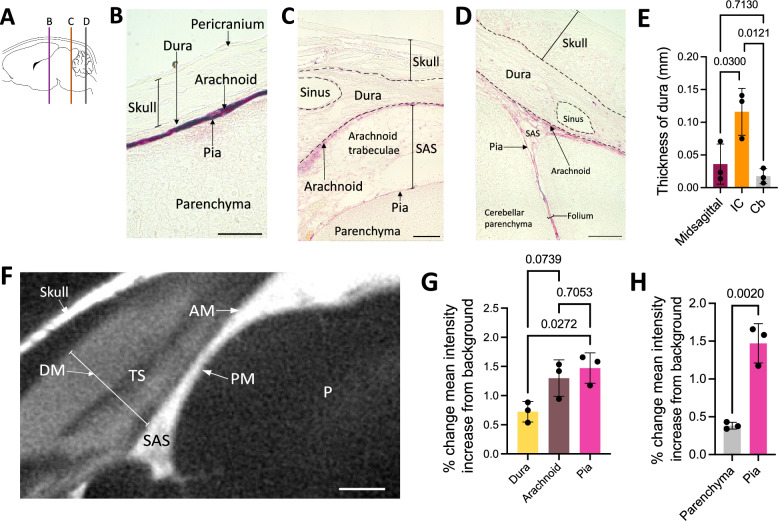
Large CSF tracers primarily circulate within the neonatal arachnoid and pia maters and not the dura. **A** Schematic of regions shown in **B**–**D**. **B**–**D**, Representative histology of decalcified skulls and the underlying meninges and parenchyma over the cortex (**B**), inferior colliculus (**C**), and cerebellum (**D**) showing minimal large CSF tracer (magenta) distribution through the pericranium, skull, and dura mater, but widespread distribution within the arachnoid and pia maters 4 h after 15-nm gold nanoparticle (AuNP) injection into the right lateral ventricle of P7 rats. There was also limited AuNP influx into the parenchyma. The subarachnoid space (SAS) is collapsed post-mortem and differentiation between layers was primarily based on tissue morphology. scalebars = 50 µm. **E** Quantification of the thickness of the midsagittal dura over the longitudinal fissure, the inferior colliculus (IC), and cerebellum (Cb). Data are mean ± SD, n = 3 per group; One-way ANOVA with post-hoc Tukey. **F** Representative X-ray microtomography (XRM) image showing high amounts of AuNP enhancement within the arachnoid mater (AM), pia mater (PM), and SAS, with less enhancement in the dura mater (DM). There was minimal enhancement in the lumen of the transverse sinus (TS) and parenchyma (P). scalebar = 500 µm. **G**, **H** Quantification of the percent change in mean intensity increase of 15-nm AuNPs in the dura, arachnoid, and pia maters (**G**) and the pia mater and parenchyma (**H**) compared to the background XRM signal. Data are mean ± SD, n = 3 per group; **G** One-way ANOVA with post-hoc Tukey. **H** Unpaired, two-tailed t-test All data are representative of 3 rodents

**Fig. 2:**
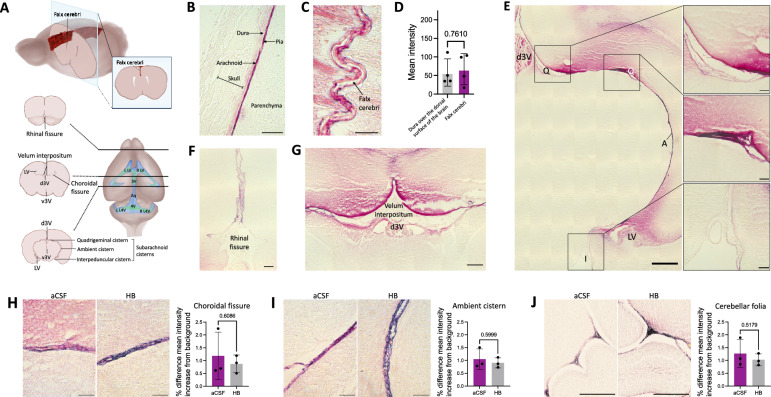
Distribution of large CSF tracers within the intracranial meninges. **A** Schematic showing location of meningeal, cerebral ventricular, and choroid plexus structures highlighted in **B**–**I**. **B** Representative histology of the meninges at the dorsal surface of the brain (including the dura, arachnoid, and pia maters) and the overlying decalcified skull 4 h after 15-nm gold nanoparticle (AuNP) injection into the right lateral ventricle of P7 rats. scalebar = 50 µm. **C** Representative histology showing 15-nm AuNP distribution within the falx cerebri and leptomeningeal (pia and arachnoid) invaginations into the longitudinal fissure. The falx cerebri is labeled with a black arrow. scalebar = 25 µm. **D** Quantification of the mean intensity of 15-nm AuNP distribution within the dura mater over the surface of the brain compared to the falx cerebri. Data are mean ± SD, n = 3 per group; Unpaired, two-tailed t-test. **E** Representative histology showing 15-nm AuNP circulation through the dorsal third ventricle (d3V), quadrigeminal cistern (Q), ambient cistern (A), interpeduncular cistern (I), and lateral ventricle (LV) 4 h after intraventricular injection in P7 rodents. E scalebar = 500 µm, E inset scalebars = 75 µm. **F**, **G** 15-nm AuNP distribution in the rhinal fissure (**F**) and velum interpositum (**G**). VI: velum interpositum; d3V: dorsal third ventricle. F scalebar = 100 µm, G scalebar = 250 µm. **H**–**J** 15-nm AuNP distribution in the choroidal fissure (**H**), ambient cistern (**I**), and cerebellar folia (**J**) 4 h post-15-nm AuNP injection into the right lateral ventricle of rats with intraventricular hemorrhage-posthemorrhagic hydrocephalus (IVH-PHH, induced 72 h before AuNP injection with intraventricular hemoglobin (HB)) and aCSF control rats. **H**, **I** scalebars = 50 μm, **J** scalebars = 250 μm. Quantifications are also shown as mean ± SD with unpaired two-tailed t tests, n = 3 animals per group. All data are representative of 3 rodents

**Fig. 3:**
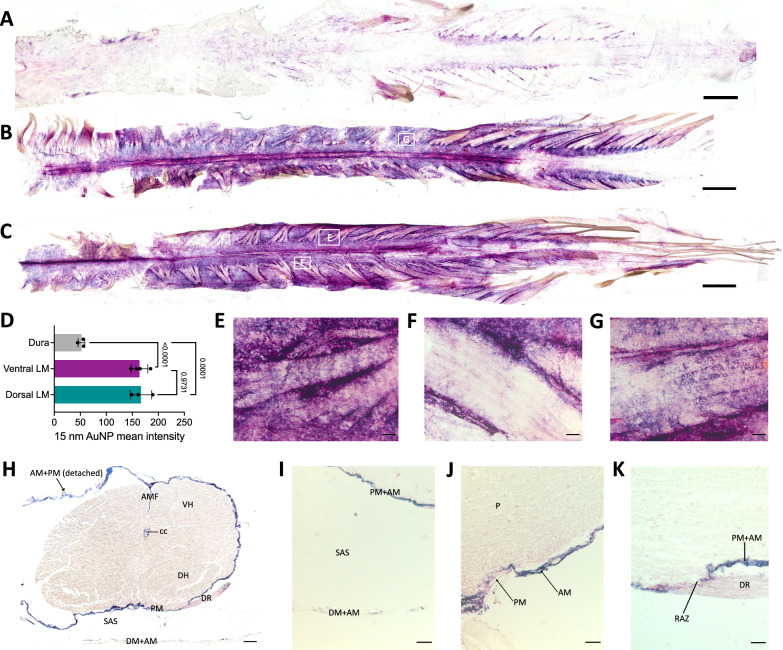
Large CSF tracers preferentially circulate within the spinal leptomeninges with limited entry superiorly into the spinal dura and inferiorly into the underlying parenchyma. **A**, **B** Representative wholemounts showing large CSF tracer distribution through the dorsal spinal cord dura (**A**) and dorsal spinal cord leptomeninges (pia and arachnoid) (**B**) 4 h after 15-nm gold nanoparticle (AuNP) injection into the right lateral ventricle of P7 rodents. Scalebar = 1 cm. **C** Representative wholemount showing 15-nm AuNP distribution through the ventral spinal cord leptomeninges 4 h after intraventricular injection into the right lateral ventricle of P7 rodents. Scalebar = 1 cm. **D**, Quantification of 15-nm AuNP mean intensity in the spine dura, ventral leptomeninges, and dorsal leptomeninges. There was significantly more 15-nm AuNP distribution in the leptomeninges than the dura. Data are mean ± SD, n = 3 per group; One-way ANOVA with post-hoc Tukey. **E**–**G** High magnification histology of 15-nm AuNP distribution along the leptomeninges of spinal nerve roots as they leave the spinal cord. 15-nm AuNPs distributed primarily around the roots (**F**), with additional diffuse 15-nm AuNPs observed inside the roots (**E**, **G**). Areas from which high-magnification images are obtained are indicated by white boxes in 2B and 2C. **E**–**G** scalebars = 25 µm. **H**–**K** Representative histology showing 15-nm AuNP distribution in the spinal cord, spinal leptomeninges and spinal dura mater. The anterior median fissure (AMF), central canal (cc), dorsal horn (DH), dorsal root (DR), dura mater (DM), arachnoid mater (AM), pia mater (PM), ventral horn (VH), subarachnoid space (SAS), root attachment zone (RAZ), and parenchyma (P) are indicated. **H** scalebar = 1 mm, **I**–**K** scalebars = 25 µm. All data are representative of 3 rodents

**Fig. 4:**
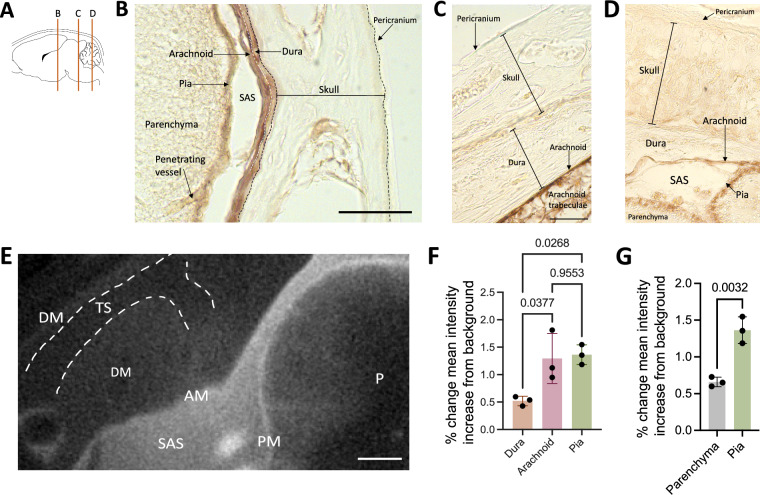
Small CSF tracers primarily circulate within the leptomeninges and not the dura. **A** Schematic of regions shown in **B**–**D**. **B**–**D** Representative histology of decalcified skulls and the underlying meninges and parenchyma showing widespread small CSF tracer distribution within the arachnoid and pia maters over the cortex (**B**), inferior colliculus (**C**), and cerebellum (**D**) 10 min after 1.9-nm gold nanoparticle (AuNP) injection into the right lateral ventricle of P7 rats. There was minimal 1.9-nm AuNP (brown) distribution through the pericranium and skull, and limited tracer distribution through the dura mater along the surface of the brain. The subarachnoid space is collapsed post-mortem and differentiation between layers was primarily based on tissue morphology. scalebar = 50 µm. **E** Representative X-ray microtomograph (XRM) showing 1.9-nm AuNP distribution within the pia mater (PM), arachnoid mater (AM), subarachnoid space (SAS), and parenchyma (P) at the level of the inferior colliculus. Similar to observations on histology, there was minimal enhancement in the dura mater (DM) and little to no enhancement in the transverse sinus (TS, dashed white line). scalebar = 500 µm. **F**, **G** Quantification of the percent change in mean intensity increase of 1.9-nm AuNPs in the dura, arachnoid, and pia maters (**F**) and pia mater and parenchyma (**G**) compared to the background XRM signal. Data are mean ± SD, n = 3 per group; **F** One-way ANOVA with post-hoc Tukey. **G** Unpaired, two-tailed T test. All data are representative of 3 rodents

**Fig. 5:**
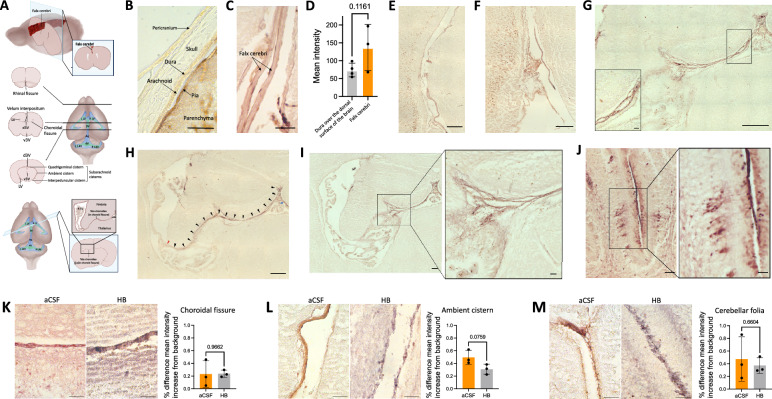
Distribution of small CSF tracers within the intracranial meninges. **A** Schematic showing location of meningeal, cerebral ventricular, and choroid plexus (ChP) structures highlighted in **B**–**L**.** B** Representative histology showing small CSF tracer (brown) distribution through the decalcified skull, underlying meninges (dura, arachnoid, and pia maters), and parenchyma 10 min after 1.9-nm gold nanoparticle (AuNP) injection into the right lateral ventricle of P7 rodents. scalebar = 50 µm. **C** Representative histology showing 1.9-nm AuNP distribution within the meningeal invaginations into the longitudinal fissure. The falx cerebri is labeled with a black arrow. C scalebar = 100 µm. **D** Quantification of the mean intensity of 1.9-nm AuNP distribution within the dura mater over the surface of the brain compared to the falx cerebri. Data are mean ± SD, n = 3 per group; Unpaired, two-tailed t-test. **E**–**G** Representative histology showing 1.9-nm AuNP distribution within the intracranial meninges of the ambient cistern (**E**), interpeduncular cistern (**F**), and choroidal fissure (**G**). **E** scalebar = 250 µm, **F** scalebar = 250 µm, **G** scalebar = 250 µm. **H**, **I** Representative histology showing the path the tela choroidea takes through the choroidal fissure from the roof of the third ventricle to the ChP of the lateral ventricle (**H**) (black arrowheads), and the tela choroidea pia mater as it gives rise to the right lateral ventricle ChP (**H**, **I**) (red arrowhead, **H**; inset **I**). **H** scalebar = 250 µm; **I** scalebar = 100 µm, **I** inset scalebar = 50 µm. **J** Representative histology of 1.9-nm AuNP distribution in the leptomeningeal invaginations into the folia of the cerebellum. **J** scalebar = 25 µm, J inset scalebar = 10 µm. **K**–**M**, 1.9-nm AuNP distribution in the choroidal fissure (**K**), ambient cistern (**L**), cerebellar folia (**M**), 10 min post-1.9-nm AuNP injection into the right lateral ventricle of rats with intraventricular hemorrhage-posthemorrhagic hydrocephalus (IVH-PHH, induced 72 h before AuNP injection with intraventricular hemoglobin (HB)) and aCSF control rats. **K**–**M** scalebars = 50 μm, **J** scalebars = 250 μm. Quantifications are also shown as mean ± SD with unpaired two-tailed t tests, n = 3 animals per group. All data are representative of 3 rodents

**Fig. 6:**
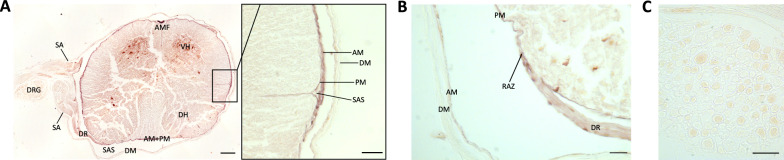
Small CSF tracers predominantly circulate within the leptomeninges of the spinal cord and parenchyma. **A**, **B** Cross sectional histology showing small CSF tracer distribution (brown) in the spinal cord parenchyma, spine leptomeninges, and spine dura 10 min after 1.9-nm gold nanoparticle (AuNP) injection into the right lateral ventricle in P7 rodents. The anterior median fissure (AMF), ventral horn (VH), subarachnoid angle (SA), dorsal root ganglion (DRG), dorsal root (DR), pia mater (PM), subarachnoid space (SAS), dura mater (DM), root attachment zone (RAZ), and arachnoid mater (AM) are indicated. A scalebar = 1 mm, **A** inset scalebar = 25 µm, B scalebar = 25 µm. **C** High magnification histology showing 1.9-nm AuNP distribution in the dorsal root ganglion. Scalebar = 50 µm. All data are representative of 3 rodents

**Fig. 7:**
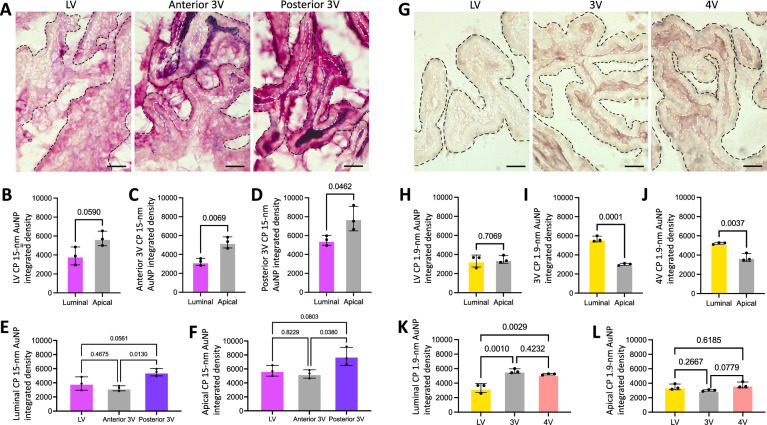
CSF tracer distribution within the choroid plexus. **A** Representative histology of large CSF tracer distribution in the lateral ventricle (LV), anterior third ventricle (3V), and posterior 3V choroid plexuses (ChP) 4 h post-15-nm gold nanoparticle (AuNP) injection into the right LV of P7 rodents. Black dotted line indicates the apical surface of ChP epithelial cells, white dotted line indicates the basolateral surface of ChP epithelial cells. Scalebars = 25 µm. **B**–**D** Quantification of 15-nm AuNP integrated density along the luminal and apical surfaces of the LV (**B**), anterior 3V (**C**), and posterior 3V (**D**) ChP. 15-nm AuNPs distributed along the apical surface of the anterior and posterior 3V ChP significantly more than the luminal surface. Data are mean ± SD, n = 3 per group; Unpaired two-tailed t test. **E**–**F** Quantification of 15-nm AuNP distribution in the LV, anterior 3V, and posterior 3V ChP along the luminal (**E**) and apical (**F**) surfaces. There was significantly more 15-nm AuNP distribution on both the luminal and apical surfaces of the posterior 3 V ChP compared to the anterior 3V ChP (**F**). Data are mean ± SD, n = 3 per group; One-way ANOVA with post-hoc Tukey. All data are representative of 3 rodents. **G** Representative photomicrographs of small CSF tracer (brown) distribution in the LV, 3V, and fourth ventricle (4V) ChP 10 min post-1.9-nm gold nanoparticle AuNP injection into the lateral ventricle in P7 rodents. Black dotted line indicates the apical surface of ChP epithelial cells, white dotted line indicates the basolateral surface of ChP epithelial cells. Scalebars = 25 µm. **H**–**J** Quantification of 1.9-nm AuNP integrated density between the luminal and apical surfaces of the LV (**H**), 3V (**I**), and 4V (**J**) ChP. There was significantly more AuNP distribution along the luminal surface of the 3V and 4V ChP compared to the apical surface. Data are mean ± SD, n = 3 per group; Unpaired two-tailed t test. **K**, **L** Quantification and comparison of 1.9-nm AuNP distribution between the LV, 3V, and 4V ChP along the luminal (**K**) and apical surfaces (**L**). There was significantly more 1.9-nm AuNP distribution throughout the luminal 3V and 4V ChP compared to the LV, but no differences between regions on the apical ChP. Data are mean ± SD, n = 3 per group; One-way ANOVA with post-hoc Tukey. All data are representative of 3 rodents

**Fig. 8:**
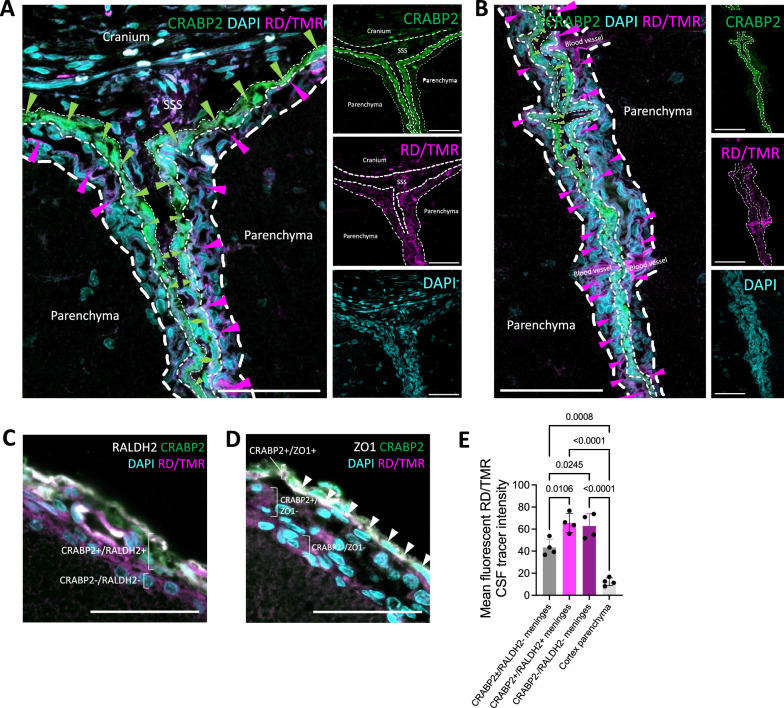
Immunofluorescent co-localization of small fluorescent CSF tracers with meningeal fibroblast markers. **A**, **B**, Representative photomicrograph of 3 kDa Red Dextran Tetramethylrhodamine (RD/TMR) (magenta, magenta arrowheads) distribution along the meninges around (**A**) and within (**A**, **B**) the longitudinal fissure 10 min after intraventricular injection into the right lateral ventricle in P7 rodents. RD/TMR distribution is primarily within the meningeal layer(s) inferior to a cellular retinoic acid binding protein 2 (CRABP2) (green, green arrowheads)-positive layer (inferior layer identified as the pia mater), with additional RD/TMR co-localization with the CRABP2 + layer (identified as the arachnoid mater). The putative border between the pia mater and underlying parenchyma is indicated with thick white dashed line; the pia and arachnoid are separated by the medium white dashed line, and the arachnoid and dura (falx) are separated by the thin white dashed line. Very little RD/TMR is seen medial/superior to the CRABP2 + layer (dura mater). **A**, **B** scalebars = 50 µm. **C**, **D** Representative photomicrographs of RD/TMR distribution in the meninges over the dorsal surface of the brain and CRABP2, retinaldehyde dehydrogenase 2 (RALDH2), and zonula occludens 1 (ZO-1) staining. RD/TMR co-localized with a CRABP2+/RALDH2+ layer (arachnoid mater), in addition to the inferiorly adjacent CRABP2-/RALDH2- layer (pia mater) (**C**). RD/TMR distribution was inferior to the CRABP2+/ZO-1 + layer (arachnoid barrier cell layer), suggesting it localizes to the arachnoid and pia maters (**D**). **E**, Quantification of RD/TMR mean intensity in CRABP2±/RALDH2− meninges (dura), CRABP2+/RALDH2+ meninges (arachnoid), CRABP2−/RALDH2− meninges (pia), and the parenchyma of the cortex. There was significantly less RD/TMR in the dura and cortex parenchyma compared to the pia and arachnoid. Data are mean ± SD, n = 4 per group; One-way ANOVA with post-hoc Tukey. All data are representative of 4 rodents

Anesthetized naïve (Figs. 1B–D, 2B, 4B–D, 5B, 8), aCSF control (Figs. 1F, 2C–G, 3, 4E, 5C–J, 6, 7), and IVH-PHH (Figs. 2H–J, 5K–M) P7 rats underwent intraventricular injection of 20 µl of 3 kDa Red Dextran Tetramethylrhodamine (RD/TMR) (D3307, Thermo Fisher Scientific, Waltham, MA), 1.9-nm (1102, Nanoprobes, Yaphank, NY), or 15-nm (1115, Nanoprobes, Yaphank, NY) gold nanoparticles (AuNPs) as per the protocol above with the following coordinates from bregma: 1.7 mm lateral, 0.5 mm anterior, and 2.0 mm deep. 1.9- and 15-nm AuNPs were constituted in aCSF at a concentration of 200 mg/mL, while RD/TMR was dissolved in aCSF in a 0.25% w/v solution. Rodents were deeply anesthetized with isoflurane and sacrificed with intracardial perfusion with 10 mL of ice-cold PBS followed by 10 mL of 4% PFA at 10 min after 1.9-nm AuNP and 3 kDa RD/TMR injections or 4 h after 15-nm AuNP injections.

### Sample preparation for meninges analysis using light microscopy

After intracardial perfusion and sacrifice, the cranium was harvested whole in a subset of rodents (Figs. 1B–D, 2B, H–J 4B–D, 5B, K–M, 8). Micro scissors were used to remove soft tissue down to the bone, before the cranium was left in 4% PFA overnight for fixation. After fixation, the cranium was rinsed in running water for 1 h and placed in 8% hydrochloric acid (HCl) or 0.12 M EDTA in PBS for 24–48 h for decalcification. After decalcification, the cranium was rinsed in running water for 2 h. Rodents that did not undergo decalcification of the cranium/spine (Figs. 2C–G, 3, 4E, 5C–J, 6, 7) had their brain/spinal cord harvested immediately after perfusion and were placed in 4% PFA overnight for fixation.

After decalcification/fixation, the cranium and/or brain was washed 2 × in PBS for 1 h each, then immersed in 30% and 50% ethanol for 30 min each, before being transferred to 70% ethanol for 24 to 72 h at 4 ºC prior to xylene and paraffin processing. The cranium/brain was embedded in paraffin, and 8–12 μm thick slices were sectioned in the coronal planes using a microtome. For sections of the tela choroidea shown in Fig. 5, sections were obtained at a 15 degree angle from coronal to show the course of the tela choroidea from the roof of the third ventricle (3V) to the LV. Slides were incubated overnight at 60 ºC before sections were soaked in xylene for 20 min and mounted with Permount mounting medium (#SP15-100, Thermo Fisher Scientific, Waltham, MA) for light microscopy localization of AuNPs, or proceeding with immunohistochemistry for immunostaining and fluorescent microscopy co-localization of RD/TMR with meningeal fibroblast markers.

### Immunohistochemistry of RD/TMR crania

After xylene, sections were rehydrated with immersion in 100%, 95%, 70%, 50%, and 30% ethanol for 5 min each. After 5 min in deionized H2O, heat-mediated antigen retrieval was performed with 10 × Diva DeCloaker (DV2004MX, BioCare Medical, Pacheco CA). Slides were cooled to room temperature before being rinsed with 1 × PBS 6 times for 5 min each and blocked in goat serum (5% normal goat serum, 2.5% BSA, 0.5% TX-100) (S-1000-200, Vector Laboratories, Newark, CA) for 1 h. Sections were incubated overnight at 4 ºC in the following primary antibodies diluted in TBST: 1:200 dilution of anti-CRABP2 (MAB5488, EMD Millipore/Sigma Aldrich, St. Louis MO), 1:200 dilution of anti-RALDH2 (#13951-1-AP, Proteintech/Sigma Aldrich, St. Louis MO), 1:100 dilution of anti-ZO-1 (ab96587, Abcam, Cambridge MA). After incubation, sections were rinsed in PBS 6 times for 5 min each followed by incubation for 60 min at room temperature with the appropriate secondary antibodies diluted in TBST: 1:2000 dilution of goat anti-mouse IgG Alexa Fluor 488 (#A-32723, Thermo Fisher Scientific, Waltham MA), 1:2000 dilution of goat anti-rabbit IgG Alexa Fluor 647 (#A-21244, Thermo Fisher Scientific, Waltham MA). Secondary antibodies were washed off with PBS 5–6 times for 5 min each before incubation with a 1:500 dilution (in PBS) of 5 mg/mL DAPI for 5 min in the dark at room temperature. Sections were washed with PBS 3–5 times for 5 min each and mounted with ProLong Gold Antifade Mountant (#P36930, Thermo Fisher Scientific, Waltham MA). Confocal images were taken with the 20× and 40× (oil) lenses on a Zeiss LSM 880 Airyscan inverted two-photon microscope (Carl Zeiss Imaging, White Plains, NY).

### AuNP quantifications from light microscope images

15-nm AuNPs appear magenta on gross visualization/light microscopy, while 1.9-nm AuNPs appear brown. Quantifications in Figs. 2D, 3D, 5D, and 7 were obtained by first opening photomicrographs in FIJI (version 2.3.0/1.53q). Images were converted to an 8-bit image type before inverting the color. Five random areas per image were analyzed for mean intensity and averaged. For quantification of the ChP (Fig. 7), the apical and basolateral surfaces of the ChP epithelial cells were identified by morphology. Averaged values were compared with statistical analyses; for comparisons with 2 groups (7B-D, 7H-J), unpaired, two-tailed t tests were performed. For comparisons with 3 groups (7E-F, 7K-L), one-way ANOVAs with post-hoc Tukeys were performed. Quantifications of dural thickness in Fig. 1E were performed by measuring the straight-line distance from the outer surface of the dura to the inner surface of the dura on XRM images in FIJI.

### RD/TMR quantifications

RD/TMR tracer distribution in Fig. 8 was quantified in FIJI by selecting 3 random regions from each layer of tissue that was identified in Fig. 8E (CRABP2+/RALDH2+, CRABP2−/RALDH2−, CRABP2±/RALDH2-, cortex parenchyma) and measuring the mean intensity of fluorescence on the magenta/red channel in FIJI. The three regions were averaged to obtain one measurement per layer of tissue per animal. This was repeated across 4 separate animals. Averaged values were compared with statistical analyses.

### Sample preparation for meninges analysis using gold nanoparticle-enhanced X-ray microtomography

After intracardial perfusion sacrifice 10 min post-1.9-nm AuNP injection and 4 h post-15-nm AuNP injection, the entire rodent body was placed in 4% paraformaldehyde at 4° C overnight, embedded in 2% agarose, and imaged within 72 h of sacrifice with a Zeiss Versa 520 X-ray microscope (Carl Zeiss Imaging, White Plains, NY) using a 0.4 × flat panel detector as previously described [37]. The X-ray source was tuned to 50 kV at 4W to optimally excite gold particles. Approximately 1600 projections were acquired and reconstructed, and tomograms were visualized in Zeiss XM3DViewer 1.2.8 (Carl Zeiss Imaging, White Plains, NY). Processed images were systematically reviewed in FIJI (Version 2.3.0).

### AuNP quantifications from XRM images

For standardization, processed images from 1.9-nm and 15-nm AuNP-XRM scans were numbered by their location relative to bregma so the location of the selected images was consistent across rodents. One image per rodent (3 rodents total for 1.9-nm AuNP-XRM and 3 rodents total for 15-nm AuNP-XRM) at the level of the inferior colliculus was selected for quantification in Figs. 1G, H and 4F, G and the corresponding XRM raw data file was opened in FIJI (version 2.3.0/1.53q). The mean gray intensity of three random regions within each anatomic ROI (dura, arachnoid, and pia) were calculated using FIJI/ImageJ and averaged to obtain one mean gray intensity measurement per anatomic ROI per rodent. The mean gray intensity of the agarose background was also obtained for each image and the percent change between the mean intensity of the anatomic ROIs and the mean intensity of the agarose background was calculated for standardization. A similar protocol was followed for the quantifications shown in Figs. 2H–J and 5K–M, however the XRM raw data files were manually selected for each region.

To quantify AuNP intensity in the pia, we selected regions of interest adjacent to the brain tissue (region of very low to no enhancement) and subjacent to the SAS (region of very high enhancement). To quantify the AuNP intensity in the arachnoid, we selected regions of interest adjacent to the SAS (region of very high enhancement) and subjacent to the dura (region of medium intensity enhancement). As a general guide to distinguish the SAS from the tissue layers of the pia and arachnoid maters, the pia mater was primarily identified as a thin rim of slightly brighter (relative to the SAS) enhancement that closely approximated the brain parenchyma tissue, and the arachnoid was identified as a thin rim of slightly brighter (relative to the SAS, but less bright than the pia) enhancement superior to the SAS. When there was more ambiguity and the pia and arachnoid could not be identified on the basis of brighter enhancement relative to the SAS, we scrolled through individual serial slices of the XRM image so that we could follow the course of blood vessels and nerves and identify a “break” or invagination in the pia as they entered/exited the brain parenchyma into the SAS, or a “break” or invagination in the arachnoid as the blood vessels and nerves exited/entered the SAS to the dura. We could subsequently follow these invaginations to identify the layers of tissue which form the invaginations to identify the pia and arachnoid respectively.

### Meningeal wholemounts

4 hours  post-15 nm AuNP injection and 10 minutes post-1.9 nm AuNP injection as previously described, rodents were deeply anesthetized with isoflurane and perfused with 10 mL of ice-cold PBS followed by 10 mL of 4% paraformaldehyde at 4 ºC. The cranium and spinal column were removed from the animal and severed at the brainstem. The cranial dura was removed by adapting a previously reported protocol [10, 18]. Curved micro scissors were inserted into the foramen magnum and used to remove the top of the skull without damaging the underlying pia and brain tissue. The skullcap and brain were separately incubated in 4% PFA overnight. The dura was dissected from the bone while the leptomeninges (pia and arachnoid maters) were carefully peeled away from the brain surface.

The spinal cord was isolated from the vertebrae by using curved micro scissors to sever the pedicles bilaterally before removing the spinal cord with forceps. The dorsal half of the spinal cord was secured with forceps and its adherent dura, which appeared as a loose-hanging translucent layer, was gently peeled away and mounted on a glass slide. The dorsal leptomeninges were gently peeled away from the underlying parenchyma. This was repeated on the ventral half of the spinal cord. Wholemounts were transferred onto a glass slide, air dried, and mounted with Permount mounting medium (#SP15-100, Thermo Fisher Scientific, Waltham, MA).

### Statistical analysis

Statistical methods were not used to recalculate or predetermine sample sizes. Associations between two continuous variables were assessed using an unpaired T-test, and associations between more than two continuous variables were assessed using a one-way ANOVA with post-hoc Tukey. All tests were 2-tailed, and p-values of less than 0.05 were considered statistically significant with all p values displayed on the graphs or reported in the corresponding figure legend. All analyses were performed using Microsoft Office Excel (Version 16.36) or GraphPad Prism (Version 9.0.0).

## Results

### Transport of large CSF solutes: brain surface

We examined differential CSF handling of large CSF tracers by the cranial leptomeninges (pia and arachnoid maters) and pachymeninges (dura mater) 4 h after intraventricular injection of 15-nm AuNPs, which appear magenta on gross visualization and light microscopy. Similar to the findings in previously published analyses of tracer distribution in the dura [10], 15-nm AuNP circulation within the dura, which was morphologically identified as the layer(s) immediately inferior to the skull, was restricted to the parasagittal and transverse sinus regions with only small amounts of 15-nm AuNP distribution in non-sinus regions (Fig. 1B–D, Additional file 1: Fig. S1A). At midline, the dura mater over the inferior colliculus was significantly thicker than the dura mater over the cortex more anteriorly and the cerebellum posteriorly (Fig. 1E).

In contrast to the dura, 15-nm AuNPs distributed uniformly throughout the leptomeninges (Fig. 1B–D). When the leptomeninges were removed, the underlying brain surface had minimal AuNP distribution, with only punctate evidence of AuNPs around the penetrating blood vessels (Additional file 1: Fig. S1B). Leptomeningeal wholemounts of the area immediately adjacent to the middle cerebral artery (MCA) supported our histologic observations of 15-nm AuNP distribution within the leptomeningeal tissue (Additional file 1: Fig. S1B).

We also obtained high-resolution whole-brain images using X-ray microtomography (XRM), which allowed for ex-vivo visualization of the meninges without dissection (Fig. 1F). Similar to what we qualitatively observed with wholemounts and histology, XRM revealed there was significantly more 15-nm AuNP distribution within the pia mater compared to the dura mater and a trend towards increased 15-nm AuNP distribution in the arachnoid mater compared to the dura mater (Fig. 1G). 15-nm AuNPs in the pia did not appear to travel inferiorly into the adjacent parenchyma (Fig. 1H), as there was significantly higher 15-nm AuNP intensity in the pia compared to the parenchyma.

### Meningeal handling of large CSF solutes within the brain

On cross section, we did not observe significant differences in 15-nm AuNP distribution between non-sinus-associated dura over the dorsal surface of the brain and the falx cerebri (Fig. 2B–D) [19]. We identified widespread 15-nm AuNP circulation through the leptomeninges of the perimesencephalic cisterns (Fig. 2E), including the quadrigeminal, ambient, and interpeduncular cisterns, and the rhinal fissure (Fig. 2F). In contrast to the meninges at the surface of the brain (Fig. 1H), there appeared to be CSF trafficking from the intracranial meninges into the adjacent parenchyma, as brain tissue adjacent to the perimesencephalic cisterns had a gradient of diffuse 15-nm AuNP distribution extending from the cisterns into the parenchyma (Fig. 2E). 15-nm AuNPs also circulated throughout the pia mater traversing the CSF spaces intracranially, including the tela choroidea in the roof of the 3 V and velum interpositum (Fig. 2G). These patterns of 15-nm AuNP distribution through the tela choroidea were similar to a previously described pattern of superficial siderosis and has implications for direct communication between the ventricles outside the ependymal-lined CSF cavities [20, 21]. There were no differences in the intensity of CSF solute distribution within the intracranial meninges after IVH-PHH (Fig. 2H–J).

### Meningeal handling of large CSF solutes within the spine

We also evaluated large CSF tracer distribution within the spine meninges. Unlike the cranial dura that adheres to the cranium during dissection, the spinal dura did not adhere to the vertebrae and was dissected away from the leptomeninges (Fig. 3A). The pia and arachnoid were peeled back from the underlying parenchyma in one piece (Fig. 3B, C).

Similar to the cranial meninges, we did not observe widespread large CSF solute distribution within the spinal dura, except for diffuse AuNPs along the lower thoracic and lumbar nerve rootlets and roots exiting the spinal cord in the lower thoracic and lumbar regions (Fig. 3A). In contrast to the minimal large CSF solute handling by the dura, we observed widespread large CSF solute circulation through the dorsal and ventral leptomeninges that outlined the structure of the median fissures and nerve roots (Fig. 3B–D). Leptomeningeal 15-nm AuNP distribution was present in higher concentrations around the nerve roots, with additional diffuse circulation within the nerve roots (Fig. 3E–G). On cross section of the spine, 15-nm AuNPs were seen in the arachnoid mater, pia mater, anterior median fissure, central canal, dorsal root, and root attachment zone (Fig. 3H–K).

### Meningeal handling of small CSF solutes on the surface of the brain and within the brain

1.9-nm AuNP-enhanced XRM in conjunction with histology was used to evaluate small CSF tracer distribution within the dura, arachnoid, and pia. 1.9-nm AuNPs appeared brown on gross visualization and light microscopy. Similar to our findings with 15-nm AuNPs, non-sinus associated dura mater had significantly less 1.9-nm AuNP distribution compared to the arachnoid and pia maters 10 min post-intraventricular 1.9-nm AuNP injection (Fig. 4A–G). While there was no overall significant difference in small CSF tracer handling between the dura over the dorsal surface of the brain and the falx cerebri, the mean intensity of 1.9-nm AuNP distribution in the falx cerebri in two out of three rodents was greater in magnitude than the mean intensity of 1.9-nm AuNP distribution in the dura over the dorsal surface of the brain in all rodents quantified (Fig. 5B–D).

We also evaluated small CSF tracer handling in the intracranial leptomeninges using histology. 10 min after intraventricular injection of 1.9-nm AuNPs, there was notable 1.9-nm AuNP circulation in the leptomeninges of the perimesencephalic cisterns, including the ambient and interpeduncular cisterns, as well as the choroidal fissure (Fig. 5E–G). This was similar to the large CSF tracer distribution patterns observed 4 h post-15-nm AuNP injection. 1.9-nm AuNPs were also observed in the pia mater of the tela choroidea in the choroidal fissure (Fig. 5G–I), and at the entry of the tela choroidea pia mater into the right LV ChP (Fig. 5I). This was akin to the 15-nm AuNP distribution seen in the dorsal 3V and velum interpositum. There was also 1.9-nm AuNP circulation in the pia mater in the folia of the cerebellum (Fig. 5J). No significant differences in small CSF tracer distribution within the intracranial meninges were observed after IVH-PHH (Fig. 5K–M).

### Meningeal handling of small CSF solutes within the spine

Similar to what we observed with large CSF solutes, there was broad small CSF solute distribution through the spinal leptomeninges 10 min after intraventricular injection, but not within the dura mater (Fig. 6A). On cross section, 1.9-nm AuNPs were concentrated in the anterior median fissure, ventral horn, dorsal root, and root attachment zone (Fig. 6A, B). 1.9-nm AuNPs were also seen within the invaginations around penetrating blood vessels (Fig. 6A). Notably, 1.9-nm AuNPs were present in the cell bodies of the dorsal root ganglia (Fig. 6A, C); the distribution among the cell bodies was not homogenous, with some cell bodies taking up more AuNPs than others (Fig. 6C).

### CSF solute handling by the choroid plexus

Given the close developmental relationship between the tela choroidea and ChP, we evaluated ChP handling of large and small CSF solutes. Large solutes were present in the LV, anterior 3V, and posterior 3V ChPs 4 h after intraventricular injection (Fig. 7A). There was significantly more apical than luminal 15-nm AuNP distribution in the anterior and posterior 3V ChPs, but no difference in the LV ChP (Fig. 7B–D). When comparing 15-nm AuNP distribution between the LV, anterior 3V, and posterior 3 V ChP, there was significantly more 15-nm AuNP distribution in the posterior 3V ChP compared to the anterior 3V ChP 4 h post-injection (Fig. 7E, F).

In contrast to large CSF solutes, there was significantly decreased small CSF solute (1.9-nm) distribution on the apical side in the 3V and 4V ChPs compared to the luminal side 10 min post-injection, but not in the LV ChP (Fig. 7G–J). There was also increased 1.9-nm AuNP distribution on the luminal side of the 3V and 4V ChPs compared to the LV ChP (Fig. 7K), however no difference was observed on the apical side between the three locations (Fig. 7L).

### Fluorescent CSF tracer colocalization with meningeal fibroblast markers

To verify the identity of the meningeal layers interacting with CSF tracer, we injected 3 kDa fluorescent CSF tracer RD/TMR into the right LV to allow for tracer co-localization with meningeal fibroblast markers including the arachnoid fibroblast markers retinaldehyde dehydrogenase 2 (RALDH2) [13], the arachnoid and dural fibroblast marker cellular retinoic acid binding protein 2 (CRABP2) [13], and the tight junction protein zonula occludens 1 (ZO-1). RD/TMR co-localized with layers of cells that were strongly positive for CRABP2 and RALDH2, and another layer that was CRABP2−/RALDH2− located superior to the brain parenchyma but inferior to the CRABP2+/RALDH2+layer (Fig. 8A–C). Both of these layers were inferior to a CRABP2+/ZO-1 layer (Fig. 8D), suggesting they represent the arachnoid and pia maters. Within cross sections of the falx cerebri, there were additional layers of tissue medial to the strongly CRABP2+ layer that largely did not have RD/TMR distribution that likely represented the dura mater (Figs. 8A, B), which is anatomically medial to the arachnoid, pia, and parenchyma in the midline. There was a small region within the dura mater that had punctate RD/TMR distribution that likely represented the superior sagittal sinus (SSS). The CRABP2+/RALDH2+ (arachnoid) and CRABP2−/RALDH2− (pia) layers had significantly more RD/TMR CSF tracer than the more superior CRABP2 ± /RALDH2- layer (dura) outside of the sinus areas (Fig. 8E), which is consistent with our observations with AuNPs that relied on morphologic identification of the meningeal layers.

## Discussion

Patterns of CSF distribution through and interaction with the cellular components of the meninges, particularly the developing intracranial leptomeninges (pia and arachnoid maters) and spine meninges are not well known [22]. In this study, we report the distribution of large and small CSF tracers within the meningeal and ventricular networks during the postnatal time period. We show that after injection into the LV, small and large CSF tracers distribute through the cranial and spinal leptomeninges, notably in the perimesencephalic cisterns and tela choroidea. In the ChP, small tracers were localized to the lumen, with differential distribution between the ventricles; the 4V and 3V ChP had significantly more tracer than the LV. Large tracers were localized to the apical ChP with significantly more tracer in the posterior 3V compared to the anterior 3V.

By closely analyzing the macroscopic distribution of CSF tracers through the meninges and ChP, we specifically hope to call attention to the role of structures outside the arachnoid granulations and dura in CSF solute distribution, especially during the neonatal and postnatal time period. The differential handling of CSF tracers by the dura and leptomeninges may suggest that the different layers of the meninges have distinct roles in CSF handling. The regional differences in small CSF tracer handing between the dura over the surface of the brain and the dura within the interhemispheric invagination (ie. the falx cerebri) in two out of three rodents suggest that the slightly higher amounts of small CSF tracer within the falx cerebri may be secondary to the falx cerebri playing a role in mediating CSF-dural sinus interactions. Specifically, the falx cerebri is in unique proximity to the superior sagittal sinus. The inferior sagittal sinus courses through the lower boundary of the falx cerebri, suggesting the falx cerebri may provide a local environment extending down the depth of the cortex in which CSF, proteins, and molecules from the interstitial fluid (ISF) and brain parenchyma are able to be trafficked into the parasagittal meningeal lymphatics to allow for ongoing CSF handling and immune monitoring, particularly in older animals with functional meningeal lymphatics [10, 23–28]. Further investigations should focus on the nature and rate of CSF transport including tracer, protein, and other molecules into the falx compared to other dural regions.

The perimesencephalic cisterns are filled with cerebrospinal fluid, surround the midbrain, and include the ambient, crural, interpeduncular, and quadrigeminal cisterns [29]. The lack of significant changes in leptomeningeal transport of CSF solutes within and around these cisterns after IVH-PHH may have implications for meningeal handling and transport of neurotoxic blood breakdown products that are released into the CSF after hemorrhage (including hemoglobin, heme, and iron [17]), and other blood products such as platelets, fibrin, and thrombin which have also previously been linked to brain injury after IVH [30–33]. Hemoglobin and iron specifically are known to be necessary for the pathogenesis of PHH after IVH [17], and preservation of meningeal CSF solute pathways may allow for transport of iron to distant locations of the brain via the perimesencephalic and other cisterns. The perimesencephalic cisterns separate two functionally-distinct regions—the hippocampus and the midbrain. This, in conjunction with the results of our present study showing diffuse 15-nm AuNP movement into only the hippocampal side of the quadrigeminal, and ambient cisterns, but not the midbrain, suggest that these cisterns may serve to facilitate a regionalized CSF-brain parenchyma cross talk [34–37]. Alternatively, their location in between the LV and 3V may serve as a potential route for inter-ventricular CSF transport and communication [21]. While we did not see any significant differences in tracer distribution within the meninges of these cisterns after IVH-PHH, it is possible there are functional differences allowing increased/decreased CSF solute transport from the cisterns into the adjacent parenchyma that should be explored in future studies. Additionally, the meninges actively mature during postnatal development, undergoing various molecular, structural, and functional changes [13, 38–42]. Future studies should explore the effects of inducing IVH-PHH at earlier or later stages of postnatal development, as developmental stage may differentially affect the extent to which CSF solutes are able to distribute across the meninges as the meninges mature.

In addition to the perimesencephalic cisterns, one particularly intriguing yet understudied intracranial meningeal structure is the tela choroidea, a thin region of pia mater adherent to the underlying 4V ependyma [43–48]. In the 3V, the tela choroidea forms the roof of the ventricle and superimposes upon itself to form the velum interpositum, which lies between the internal cerebral veins and contains cerebrospinal fluid [43, 45, 47, 49, 50]. The 3V tela choroidea is continuous with the LV via the choroidal fissure. The tela choroidea also gives rise to the ChP in each of the brain's ventricles. The tela choroidea has been purported to have a role in allowing CSF to be recirculated into the ventricular system but has otherwise been sparsely studied in the context of CSF circulation [20, 51, 52]. In this study, we report that 1.9-nm AuNPs were found predominantly in the luminal side of the 3V and 4V ChP as soon as 10 min post-injection. There was significantly less luminal 1.9-nm AuNP in the LV ChP compared to the 3V and 4V. In addition, there was significantly less apical compared to luminal 1.9-nm AuNP distribution in all of the ventricles, suggesting that the 1.9-nm AuNPs were not being shuttled across the ChP epithelial cells into the lumen, but rather directly transported along the luminal pia mater. It is possible that the first point of 1.9-nm AuNP entry to the ChP is through the tela choroidea of the 4V or the roof of the 3V and eventually the choroidal fissure and LV ChP. A previous study on experimental hydrocephalus in primates and dogs posited that CSF traveled to the ventricles from the cisterns via the tela choroidea [52, 53]. Other studies have indicated that CSF may be transported across a normally functioning tela choroidea into the ChP and ventricles as a regular aspect of brain physiology [20, 51]. The results of this present study support these findings and build upon previous studies implicating the ChP in direct CSF solute transport [54, 55]. Future studies should map the timeline of CSF tracer distribution along the tela choroidea and LV, 3V, and 4V ChP in vivo.

While not as well-studied in the neonatal and postnatal time period, there has been a long-standing body of literature suggesting CSF tracers injected into the ventricles and cisterns of adult and aged animals are eventually drained into the lymphatic system [10, 12, 56–69]. There are several proposed routes by which this occurs: (1) perivascular and perineural routes into extracranial and extraspinal soft tissue, after which they are absorbed into the lymphatic system, and (2) dural meningeal lymphatics [10, 57]. In the latter theory, it is not entirely clear how solute movement bridges the CSF spaces (SAS) and dura (where meningeal lymphatics are located), as CSF solutes would presumably have to pass through the blood-CSF barrier posed by the outer arachnoid which separates the SAS from the dura. The results in this study suggest that CSF tracer in the SAS may not cross the arachnoid layer of the meninges to enter into the dura in great quantities, a finding which is supported by previous literature [70–75]. This finding calls the role of dural meningeal lymphatics in CSF outflow during the postnatal period into question, suggesting alternative routes of CSF outflow exist. One caveat is that the experiments in this study were conducted in postnatal rodents, a time during which meningeal lymphatics may not be fully developed [28]. Future studies in adult rodents should investigate the nature of these functional connections to elucidate the specific route by which CSF solutes are transported from the CSF into the dural meningeal lymphatics. Future studies should also investigate in parallel alternative routes of CSF outflow which may play a greater role in CSF drainage than the dural meningeal lymphatics, including CSF outflow along perivascular and perineural subarachnoid spaces into extracranial lymphatics of the skull base [12].

One limitation of this study is that our investigations were conducted ex vivo, which may impact the structure and presence of CSF spaces such as the SAS. Similarly, while decalcification with EDTA and HCl allowed for visualization of the skull, meninges, and brain in approximation with each other, it resulted in disfiguration of the skull over the dorsal convexities. Our investigation was based on rodents, animals in which the presence, morphology, and function of arachnoid villi have been debated. Additionally, the conclusions in this study were made in part on the basis of images obtained using light microscopy; future studies should examine CSF tracer distribution at an ultrastructural level in the regions we called out in this present study. Finally, the animal (rat) we used for this study is lissencephalic, which may affect the leptomeningeal distribution of CSF tracers in the cerebrum as the pia mater follows the invaginations produced in animals with gyri and sulci. In the cerebellum, which retains its sulci and gyri in the rodent brain, there was an abundance of CSF tracer in the pia mater invaginations into the folia. Repeating these experiments in organisms with gyri and sulci, such as pigs or ferrets, may show similar patterns of  tracer in the leptomeninges over the cerebral cortex.

## Conclusion

We present a CNS-wide map of meningeal handling of large and small CSF solutes in the neonatal brain and spine. Intracranial leptomeninges, particularly the tela choroidea in the 4V and at the roof of the 3V, are an important area for CSF circulation and future studies should be performed to characterize mechanisms of CSF transport in these areas. Differential ChP distribution of tracers by ventricle and between the luminal and apical surfaces suggests region-specific functions in facilitating CSF flow. Finally, we show that meningeal handling of both large and small CSF solutes is mediated primarily by the pia and arachnoid in young animals.

### Supplementary Information

**Additional file 1.**Supplementary methods and Supplementary Figure 1.

## Data Availability

The datasets generated during and/or analysed during the current study are available from the corresponding author on reasonable request.

## Abbreviations

AuNP: Gold nanoparticle
ChP: Choroid plexus
CNS: Central nervous system
CSF: Cerebrospinal fluid
ISF: Interstitial fluid
IVH: Intraventricular hemorrhage
PHH: Posthemorrhagic hydrocephalus
RD/TMR: Red dextran tetramethylrhodamine
SAS: Subarachnoid space
3V: Third ventricle
4V: Fourth ventricle
LV: Lateral ventricle
